# RGC32 induces epithelial-mesenchymal transition by activating the Smad/Sip1 signaling pathway in CRC

**DOI:** 10.1038/srep46078

**Published:** 2017-05-04

**Authors:** Xiao-Yan Wang, Sheng-Nan Li, Hui-Fang Zhu, Zhi-Yan Hu, Yan Zhong, Chuan-Sha Gu, Shi-You Chen, Teng-fei Liu, Zu-Guo Li

**Affiliations:** 1 Department of Pathology, Nanfang Hospital, Southern Medical University, Guangzhou 510515, China; 2 Guangdong Provincial Key Laboratory of Molecular Tumor Pathology, Department of Pathology, School of Basic Medical Sciences, Southern Medical University, Guangzhou 510515, China; 3 Department of Physiology & Pharmacology, University of Georgia, Athens, GA, United States

## Abstract

Response gene to complement 32 (RGC32) is a transcription factor that regulates the expression of multiple genes involved in cell growth, viability and tissue-specific differentiation. However, the role of RGC32 in tumorigenesis and tumor progression in colorectal cancer (CRC) has not been fully elucidated. Here, we showed that the expression of RGC32 was significantly up-regulated in human CRC tissues versus adjacent normal tissues. RGC32 expression was significantly correlated with invasive and aggressive characteristics of tumor cells, as well as poor survival of CRC patients. We also demonstrated that RGC32 overexpression promoted proliferation, migration and tumorigenic growth of human CRC cells *in vitro* and *in vivo*. Functionally, RGC32 facilitated epithelial-mesenchymal transition (EMT) in CRC via the Smad/Sip1 signaling pathway, as shown by decreasing E-cadherin expression and increasing vimentin expression. In conclusion, our findings suggested that overexpression of RGC32 facilitates EMT of CRC cells by activating Smad/Sip1 signaling.

Colorectal cancer (CRC) is a high-risk digestive tract tumor^1^. Prevention, early diagnosis and treatment can greatly decrease its incidence and mortality. However, doing so requires comprehensive understanding of every aspect of CRC at the molecular and the cellular level. A preponderance of evidence shows that epithelial-mesenchymal transition (EMT), characterized by down-regulation of epithelial markers and increased expression of mesenchymal markers, is involved in the metastasis of many types of cancers, including CRC^2^. Thus, molecular markers that promote EMT in CRC may play an important role in the progression of CRC.

Response gene to complement 32 (RGC32) is a novel cellular protein that was first cloned from oligodendrocytes by Badea *et al*. in 1998^3^. It plays important roles in the regulation of cell differentiation, angiogenesis, migration, and invasion^4^,^5^,^6^. In addition, emerging evidence has revealed that RGC32 is involved in lung cancer^7^, breast cancer^8^, hepatic steatosis^9^, and multiple sclerosis^10^. However, little is known about its function in CRC. Most importantly, our previous studies have demonstrated that RGC32 mediates EMT in human renal proximal tubular cells (HPTCs) by interacting with Smad3^11^,^12^. Therefore, we hypothesized that RGC32 may also participate in Smad-induced EMT in CRC.

Sip1 (Smad-interacting protein 1), also known as ZEB2, is a member of the ZEB family of zinc finger transcriptional factors. Sip1 consists of a central homeodomain, a CtBP (C-terminal-binding protein) binding domain and Smad-interacting domains, as well as two clusters of zinc fingers at both termini. Importantly, the transcriptional changes mediated by Sip1 occur through both direct and indirect binding to downstream gene promoters^13^. In addition, Sip1 contributes to cytoskeletal changes and increased cell motility^14^. Although there have been several studies regarding the role of Sip1 in early development of cell differentiation^15^,^16^,^17^,^18^,^19^,^20^, little is known about the function of Sip1 during EMT in CRC.

In this study, we report that overexpression of RGC32 is related to poor overall survival in patients with CRC. We demonstrate that RGC32 promotes cell proliferation, motility, and invasion of CRC. Furthermore, we speculate that up-regulation of the expression of RGC32 contributes to EMT in CRC by activating the Smad/Sip1 signaling pathway. Finally, we suggest that RGC32 protein, a valuable CRC prognostic marker, plays an important role in the development and progression of human CRC.

## Results

### RGC32 was upregulated and was associated with patient poor survival

**Figure 1:**
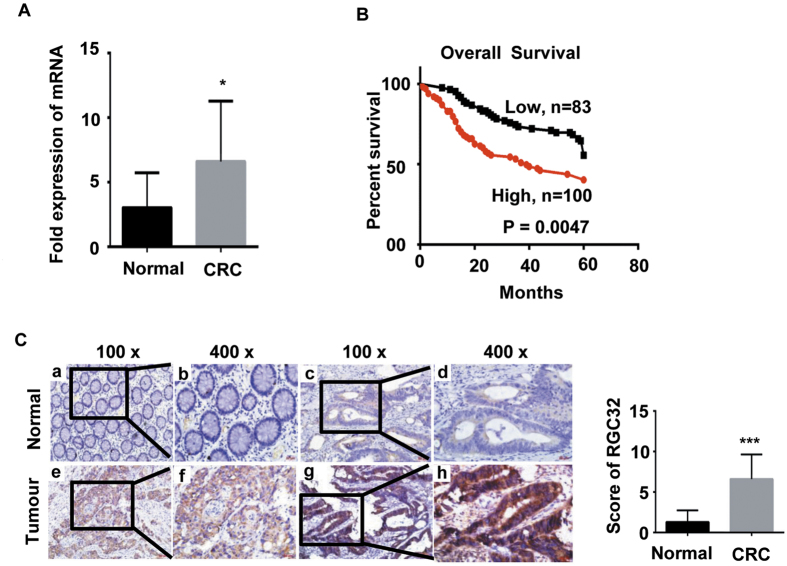
RGC32 was overexpressed and was associated with poor survival in CRC patients. (**A**) The expression of RGC32 mRNA in normal and tumor tissues of patients with CRC was detected with real-time PCR. (**B**) Kaplan–Meier analysis of overall survival in patients with CRC. (**C**) Representative images of RGC32 expression in normal intestinal epithelium and CRC specimens examined by IHC. RGC32 was not detected (a, b) in normal intestinal epithelial cells, whereas it was detected in CRC cells (c and d display weak signals, e and f display moderate signals, and g and h display strong signals). *P < 0.05, ***P < 0.001 compared with the score of RGC32 in normal tissue.

Real-time PCR analysis was used to test the expression of RGC32 in 12 paired CRC tissues and adjacent normal colorectal tissues. The results indicated that the expression of RGC32 was higher in CRC tissues (C) than in adjacent normal colorectal tissues (N) (6.952 ± 1.355 versus 3.025 ± 0.7769, P < 0.05) (Fig. 1A). IHC was used to detect the expression level of RGC32 in 183 paraffin-embedded CRC tissue samples, representing 183 CRC cases. Kaplan–Meier survival analysis showed that patients with a higher RGC32 expression level had shorter survival times than those with lower expression levels (P = 0.0047) (Fig. 1B). Furthermore, The results showed that the expression of RGC32 was markedly increased in 170/183 of the CRC tissues. The score of RGC32 expression in CRC was higher than that in normal colorectal mucosa (P < 0.001) (Fig. 1C). Moreover, analysis of the clinicopathological characteristics of all 183 tissue samples showed that RGC32 levels were closely associated with the degree of lymph node metastasis (P = 0.043), Dukes’ classification (P = 0.035) and TNM stage (P = 0.048) (Table 1).

### RGC32 promoted CRC proliferation *in vitro* and *in vivo*

RT-PCR and western blotting analysis were used to test the expression of RGC32 in 6 CRC cell lines, namely SW620, SW480, LS174T, HCT116, HT29 and SW480/M5 cell lines. Our results revealed that RGC32 was differentially expressed in all 6 CRC cell lines. SW620 and SW480/M5 cells exhibited much higher RGC32 expression levels than did other cells (Supplement Figure 1A,B). To assess the role of RGC32 in CRC cells, an RGC32-overexpressing cell line SW480/RGC32 was established (Supplement Figure 1C,D). A CCK8 proliferation assay showed that RGC32 overexpression promoted the proliferation of SW480 cells compared with the SW480/vector cells (Supplement Figure 1E; P < 0.05). To further investigate the effects of RGC32 on CRC cells, we knocked down endogenous RGC32 in SW620 cells with shRNAs specifically targeting RGC32 (Supplement Figure 1F,G). The results of the CCK8 proliferation assay demonstrated that depletion of endogenous RGC32 in SW620 cells caused a marked decrease in cell growth compared with that of control cells (Supplement Figure 1H; P < 0.05).

To confirm the effect of RGC32 on tumor proliferation *in vivo*, SW620/scramble and SW620/shRGC32 cells were subcutaneously inoculated into athymic nude mice. All mice developed xenograft tumors at the injection site. As shown in Supplement Figure 2A, SW620/shRGC32 cells implanted in nude mice exhibited a lower tumor growth rate and significantly smaller tumor volumes. In other words, knockdown of endogenous RGC32 expression in SW620 cells caused significant inhibition of tumor growth (n = 6, P < 0.05). In addition to the differences in tumor volume, we also found that the tumors formed by SW620/shRGC32 cells displayed a lower Ki-67 index than that did tumors formed by SW620/scramble cells, as detected through IHC analysis of Ki-67 (Supplement Figure 2B,C).

### RGC32 promoted the migration and invasion of CRC cell lines *in vitro*

**Figure 2:**
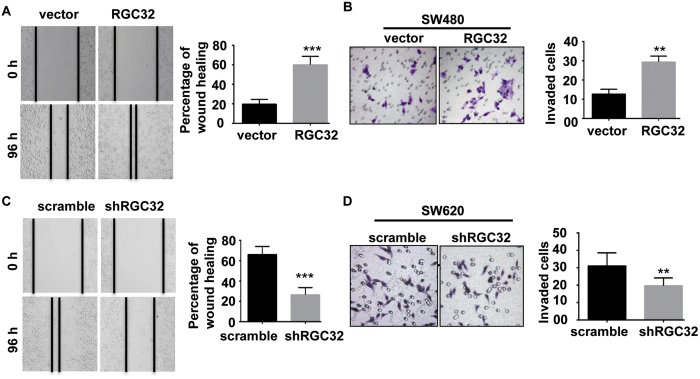
RGC32 promoted migration and invasion of CRC cell lines *in vitro*. (**A,B**) Overexpression of RGC32 markedly increased SW480 cell migration and invasion as measured with wound-healing (**A**) and with Transwell invasion assays (**B**). (**C,D**) Knockdown of RGC32 markedly decreased SW620 cell migration and invasion as measured by wound-healing (**C**) and Transwell invasion assays (**D**). **P < 0.01 vs vector or scramble; ***P < 0.001 vs vector or scramble.

We examined the effects of RGC32 on CRC cell migration and invasion. A wound-healing assay illustrated that RGC32 overexpression in CRC cells resulted in a significant increase in cell migration (P < 0.001, Fig. 2A). The results of the Matrigel invasion assay showed that overexpression of RGC32 promoted cell invasion in SW480 cells (P < 0.01, Fig. 2B). In contrast, migration and invasion were decreased by RGC32 knockdown in SW620 cells (P < 0.01, Fig. 2C,D). These results indicated that RGC32 facilitated CRC cell migration and invasion *in vitro*.

### RGC32 promoted EMT of tumor cells

**Figure 3:**
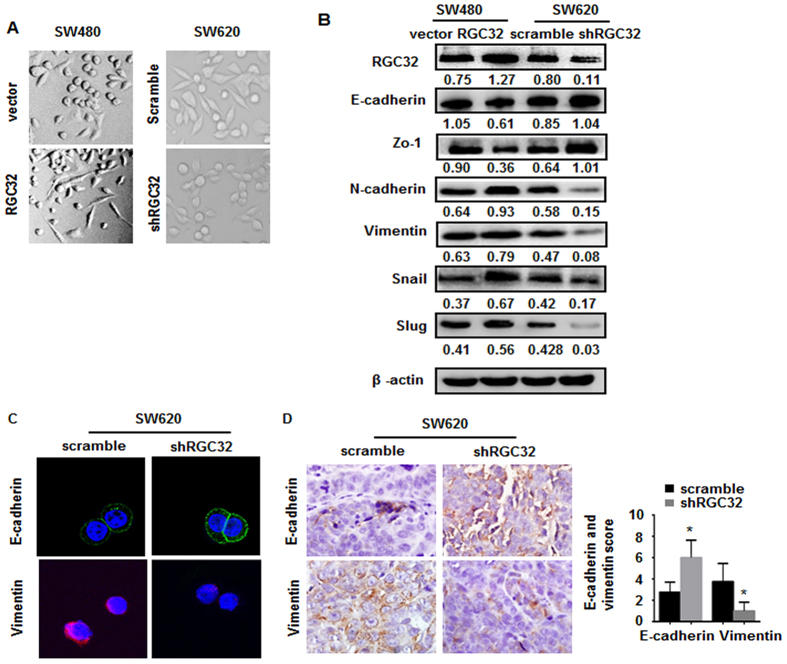
RGC32 induced EMT phenotype marker alterations in colorectal cancer. (**A**) The morphology of SW480 cells transfected with vector or RGC32 plasmid (RGC32) and SW620 cells transfected with scramble or RGC32 shRNA (shRGC32). (**B**) The expression of mesenchymal and epithelial markers in SW480 cells transfected with vector or RGC32 plasmid (RGC32) and SW620 cells transfected with scramble or RGC32 shRNA (shRGC32) was detected by western blotting. (**C**) Confocal microscopy of E-cadherin and vimentin staining in SW620 cells transfected with scramble or RGC32 shRNA(shRGC32). (**D**) Immunohistochemical staining of the EMT-related proteins E-cadherin and Vimentin in primary tumor tissues derived from scramble or RGC32-containing SW620 cells as indicated. Magnification: 400x; *P < 0.05 vs scramble.

Because the expression levels of RGC32 were correlated with CRC metastasis in humans, and RGC32 altered CRC cell morphology (Fig. 3A). SW480 cells became elongated after RGC32 overexpressed while SW620 cells got stretched by knockdown of RGC32. We hypothesized that up-regulation of RGC32 might participate in EMT in CRC cells, a process implicated in the metastasis of a large number of tumors.

To determine whether RGC32 participated in EMT in CRC cells, we detected the expression of epithelial markers (E-cadherin, occludin-1 and ZO-1) and EMT markers (N-cadherin, vimentin, snail and slug). Western blotting showed that up-regulation of RGC32 in SW480 cells significantly decreased the expression of E-cadherin and ZO-1, whereas it increased the expression of N-cadherin, vimentin, snail and slug. In contrast, knockdown of RGC32 in SW620 cells markedly increased the expression of E-cadherin and ZO-1, whereas it decreased the expression of N-cadherin, vimentin, snail and slug (Fig. 3B). The immunofluorescence analysis showed similar results (Fig. 3C). IHC staining showed that the tumors of the SW620/shRGC32 group displayed much stronger E-cadherin staining and weaker vimentin staining than did tumors in the SW620 scramble group (Fig. 3D). Therefore, these results suggested that RGC32 induces EMT in CRC cells.

### RGC32 regulated the Smad/Sip1 signaling pathway

Smad signaling is well known to play an important role in EMT. Sip1 (Smad interacting protein 1, Zeb2/ZFHX1B) has also been postulated to play an important role in EMT. In addition, Sip1 has been shown to directly bind and repress E-cadherin expression in cancer cells, thus facilitating EMT. More importantly, our previous studies have shown that RGC32 is critical for Smad-induced EMT in human renal proximal tubular cells[7]. Thus, we hypothesized that in CRC cells, the Smad/Sip1 signaling pathway may be involved in RGC32-induced EMT.

**Figure 4:**
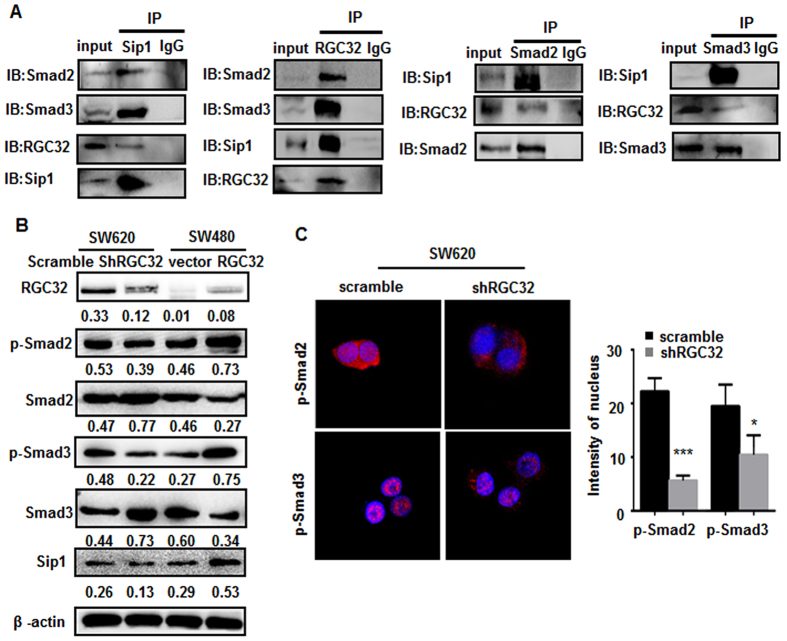
RGC32 regulated the Smad/Sip1 signaling pathway. (**A**) Coimmunoprecipitation assay to test the correlation between RGC32, Smad2/3 and Sip1. (**B**) The expression of Smad signaling and Sip1 in SW480 cells transfected with vector or RGC32 plasmid (RGC32) and SW620 cells transfected with scramble or RGC32 shRNA(shRGC32) was detected by western blotting. (**C**) The expression of p-Smad2 and p-Smad3 in cytolymph and the nucleus detected by immunofluorescence. *P < 0.05, ***P < 0.001 vs scramble.

First, to verify this hypothesis, we set up a coimmunoprecipitation assay to test the correlation between RGC32, Smad2/3 and Sip1. The results revealed that RGC32, Sip1, Smad2 and Smad3 interacted with each other (Fig. 4A). Then, to further determine the relationship between RGC32 and the Smad/Sip1 signaling pathway in CRC, we performed a western blotting assay to detect the influence of RGC32 on the expression of Sip1 and Smad proteins. The results showed that knockdown of RGC32 slightly decreased the expression of Sip1 and phosphorylation of Smad2, and Smad3. In contrast, up-regulation of RGC32 increased the expression of these proteins (Fig. 4B). The immunofluorescence staining results indicated that knockdown of RGC32 repressed the phosphorylation of p-Smad2 or p-Smad3 in the nucleus (Fig. 4C). The nucleus immunofluorescence intensity of p-Smad2 or p-Smad3 decreased in SW620 cells when knockdowned the expression of RGC32 (P < 0.05). The subcellular localization of p-Smad2 and p-Smad3 suggested that RGC32 activates the Smad/Sip1 pathway and promotes translocation of p-Smad2 and p-Smad3 into nucleus, thus inducing EMT and enhancing the metastatic ability of CRC cells.

#### The Smad/Sip1 signaling pathway mediated the RGC32-induced EMT in CRC

**Figure 5:**
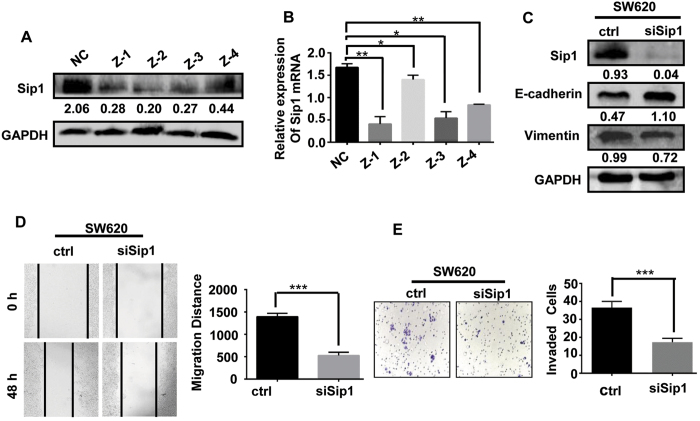
The Smad/Sip1 signaling pathway mediated RGC32-induced EMT in CRC. (**A,B**) Knockdown of endogenous Sip1 in SW620 cells shown by RT-PCR and western blotting. (**C**) The expression of mesenchymal and epithelial markers in SW620 cells transfected with scramble (Ctrl) or Sip1 siRNA (siSip1) was detected by western blotting. (**D,E**) *In vitro* scratch wound-healing assays and migration assays were performed to investigate the role of Sip1 in CRC cell migration. *P < 0.05, **P < 0.01, ***P < 0.001 vs ctrl.

To further investigate our speculation that RGC32 may regulate EMT through the Smad/Sip1 signaling pathway in CRC cells, we used siRNAs specifically targeting Sip1 to knock down endogenous Sip1 in SW620 cells (Fig. 5A,B). A western blotting analysis showed that the epithelial marker E-cadherin was up-regulated, whereas the mesenchymal markers N-cadherin and vimentin were down-regulated in Sip1-knockdown CRC cells (Fig. 5C). In addition, an *in vitro* scratch wound-healing assay and migration assay were performed to investigate the role of Sip1 in CRC cell migration. Knockdown of Sip1 indeed decreased CRC cell migration (Fig. 5D,E), thus indicating that the Smad/Sip1 signaling pathway plays an important role in CRC migration. These data demonstrated that the Smad/Sip1 signaling pathway, which is regulated by RGC32, promotes CRC cell migration through EMT.

## Discussion

RGC32 has been identified as a cell cycle regulatory factor that promotes cell proliferation, as both an activator and a substrate of p34CDC2^21^. In addition, RGC32 may be an oncogene in EBV-infected cells by promoting the survival and proliferation of cells, as well as deregulating of the G2/M cell-cycle checkpoint^22^. Although RGC32 expression has been detected in a wide range of human tumors, there is still controversy regarding the function of RGC32 in cancer development and progression, and it has also been reported that RGC32 mRNA expression is lower in advanced stages of primary astrocytomas^23^. In our study, the IHC results showed that RGC32 expression was closely correlated with lymph node status and Dukes’ classification of CRC patients (P < 0.05). Moreover, expression of RGC32 protein was a significant prognostic factor for poor overall survival in CRC patients. These findings supported the idea that RGC32 plays a key role in CRC development and suggested that RGC32 may be used as an independent predictor of prognosis for CRC patients.

Emerging evidence shows that RGC32 is associated with cancer progression. However, the molecular mechanism of how RGC32 regulates aggressive features of cancer cells is largely unknown. Some studies have reported a higher RGC32 expression level is observed in adenomas compared with normal colon tissue^24^ and have suggested that RGC32 may contribute to the development of colon cancer by regulating chromatin assembly^23^. In addition, RGC32 methylation has been found in non-small cell lung cancers (NSCLC), and methylation-associated down-regulation of RGC32 plays an important role in the pathogenesis of NSCLC^4^. Our previous studies have demonstrated that RGC32 plays a critical role in TGF-β-induced EMT of renal tubular cells^11^. The present study sought to determine whether RGC32 is essential for EMT in CRC cells. We first manipulated RGC32 expression by transfecting an RGC32-expression plasmid into CRC cells. We found that RGC32 overexpression enhanced expression of N-cadherin, vimentin, snail, slug and TCF8/ZEB1 and inhibited expression of E-cadherin and ZO-1. These results suggest that RGC32 functions as an inducer of EMT in tumor progression of CRC.

Our previous study has shown that RGC32 plays an essential role in Smad3-mediated activation of myofibroblast marker gene transcription. Sip1, also called ZEB2, is a member of the Zfh1 family of 2-handed zinc finger/homeodomain proteins^25^. It is located in the nucleus and functions as a DNA-binding transcriptional repressor that interacts with activated Smads^16^. Sip1 appears to be one of the representative epithelial–mesenchymal transition (EMT) regulators^26^,^27^,^28^ and is an EMT-inducible gene that plays a key role in tumor progression in various cancers^29^. Some researchers have found that Sip1 protein binds proximal E-boxes within the E-cadherin gene (cdh1) promoter, and CDH1 transcriptional down-regulation induces EMT in developmental processes and during tumor cell invasion and metastasis. Here, we found that RGC32 induced EMT in SW620 cells, and Sip1 protein was activated through up-regulation of RGC32. Therefore, we speculate that the Smad/Sip1 pathway participates in the process of RGC32-induced EMT.

TGF-β and its downstream signaling molecules have been shown to play an essential role in EMT. When TGF-β signaling is initiated, the activated TGF-β type I receptor phosphorylates the downstream signaling mediators Smad2 and Smad3, which then bind to Smad4 and translocate into the nucleus, where they act as transcription factors activating the expression of TGF-β response genes involved in EMT. To explore whether Smad/Sip1 signaling pathways are responsible for RGC32-induced EMT in CRC, we examined the activation of Smad proteins. There were significant differences in the expression of Smad2, p-Smad2, Smad3 and p-Smad3 between RGC32-overexpressing CRC cells and RGC32-knockdown cells. Therefore, the mechanisms underlying EMT in CRC appear to be similar to those of EMT in renal tubular cells.

In summary, our findings suggest that overexpression of RGC32 might be a valuable prognostic marker for CRC progression, and RGC32 promotes CRC cell proliferation and invasion *in vitro* and *in vivo*. RGC32 activates the Smad/Sip1 signaling pathway and modulates EMT in CRC, thus participating in the occurrence and development of CRC. Therefore, RGC32 may be used as a potential target for CRC prevention and therapy.

## Materials and Methods

### Clinical specimens and cell culture

Tumor samples were obtained from patients with pathologically diagnosed colorectal cancer between January 2001 and December 2010 at Nanfang Hospital, Southern Medical University. Clinicopathological classification of these samples was performed according to the General Rules for Clinical and Pathological Studies on Cancer of the Colon, Rectum, and Anus along with the International Union Against Cancer classification system. The use of tissues for this study was approved by the ethics committee of Nanfang Hospital, Southern Medical University. All of the patients signed informed consents before use of these clinical materials for research purposes. This study was performed in strict accordance with the recommendations in the Guide for the Care and Use of Laboratory Animals of the National Institutes of Health. The protocol was approved by the Committee on the Ethics of Animal Experiments of Southern Medical University (Permit Number: SYXK2011-0074). All surgery was performed under sodium pentobarbital anaesthesia, and all efforts were made to minimize suffering.

Human CRC cell lines (HCT116, HT29, Lovo, SW480, SW620 and LS174T) were initially purchased from American Type Culture Collection (Manassas, VA, USA). The SW480/M5 cell line was established in our laboratory. All cell lines were cultured in RPMI 1640 medium containing 10% foetal bovine serum (Gibco, USA) and were incubated in 5% CO_2_ at 37 °C.

### RNA isolation and real-time PCR

Total RNA was isolated from cells with TRIzol reagent (Takara), and cDNA was synthesized with a Reverse Transcription Kit (Takara). Quantitative real-time PCR analyses were performed in triplicate using SYBR Green I (Takara). The primers for human RGC32 were 5′-GCCACTTCCACTACGAGGAG-3′ (forward) and 5′-GTGGCCTGGTAGAAGGTTGA-3′(reverse). The primers for human Sip1 were 5′-GATGGGAAAATGGAAACCAAATCAGACCAC-3′(forward) and 5′-TTCTGTCCCTCTCTACAGCTTCCTGGAAGC-3′ (reverse). The primers for human GAPDH were 5′-ACA GTC AGC CGC ATC TTCTT-3′ (forward) and 5′-GAC AAG CTT CCC GTT CTC AG-3′ (reverse). The mRNA levels of specific genes was normalized against human GAPDH levels.

### Western blot analysis

Proteins were extracted with a lysis buffer and quantified with a quantitative bicinchoninic acid (BCA) protein assay (KeyGen Biotech). Protein lysates were separated using 10% SDS-PAGE and electroblotted onto a polyvinylidene difluoride (PVDF) membrane (Roche). The membrane was blocked with 5% bovine serum albumin for 1 h and then incubated with rabbit polyclonal antibody against RGC32 (Santa Cruz), Sip1 (Abcam), Smad2, p-Smad2, Smad3, p-Smad3, E-cadherin, N-cadherin, or vimentin (Cell Signaling Technology), or with mouse monoclonal antibody against β-actin at 4 °C overnight. The membrane was washed and incubated with secondary antibodies, and then, immunoreactive proteins were detected with an enhanced chemiluminescence (ECL) detection system (FDbio) according to the manufacturer’s instructions.

### Immunohistochemistry

To study alterations in protein expression in the 183 archived, formalin-fixed paraffin-embedded human CRC specimens, 3-μm-thick sections were mounted on aminopropylethoxysilane (APES)-treated slides and incubated for 1 h at 65 °C. After being deparaffinised with xylene and rehydrated with a graded series of ethanol to distilled water, the sections were submerged in sodium citrate antigen-retrieval buffer (pH 6.0) and microwaved. Endogenous peroxidase activity was quenched by incubation of the sections in 0.3% hydrogen peroxide with methanol. Sections were subsequently treated with 1% bovine serum albumin for 30 min to reduce non-specific binding, and this was followed by overnight incubation with antibodies against RGC32 (sc-84222, Santa Cruz) at a dilution of 1:200. After washing, the sections were incubated further with HRP at room temperature for 30 min. For the staining reactions, diaminobenzidine (DAB) was used. For negative controls, the antibody was replaced with normal goat serum.

A relatively simple, reproducible scoring method was used for the immunohistochemical evaluation of RGC32 in CRC^30^. The staining intensity was scored as 0 (negative), 1 (weak), 2 (medium), or 3 (strong). The area of positive tumor cells was graded as 0 (0%), 1 (1–25%), 2 (26–50%), 3 (51–75%), or 4 (76–100%), according to the percentages of positive-staining areas of tumor cells or normal colonic cells in relation to the entire carcinoma-involved area or, for the normal samples, the entire section. An overall protein expression score (overall score range, 0–12) was calculated by multiplying the intensity and positivity scores. For the purpose of statistical evaluation, tumors with a final staining score >5 were considered to be high-expressing.

### Immunofluorescence

For immunofluorescence staining of cultured cells, cells seeded on confocal dishes were transfected with adenoviral vectors. After 48 h, the cells were fixed with 4% paraformaldehyde for 30 min and permeabilized with 0.5% Triton X-100 for 10 min at room temperature. The cells seeded on confocal dishes were incubated with primary antibodies at 4 °C overnight and then washed with PBS and incubated with fluorescent secondary antibody in the dark at room temperature for 1 h. After being washed several times with PBS, the confocal dish was mounted using an anti-fade mounting solution containing 4,6-diamidino-2-phenylindole (DAPI). The staining was examined, and images were captured using an Olympus FV1200 Confocal Laser Scanning microscope.

### Construction of the RGC32 expression plasmid and transfection

RGC32 cDNA was amplified from mRNA extracted from normal human colon tissue. The 5′ primer included a BamHI restriction site for cloning and a Kozak sequence, followed by the RGC32 cDNA sequence. The 3′ primer included the RGC32 cDNA sequence, a stop codon, and an XbaI restriction site. For cloning, both the pcDNA3.0 vector and the amplified RGC32 cDNA were digested with BamHI and XbaI and then purified and subjected to ligation using T4 DNA ligase. The cloned cDNA was verified by sequencing. SW480 cells were transfected with the RGC32-expression plasmid by using Lipofectamine 2000 (Invitrogen). After 24 and 48 h of incubation, cellular RNA and protein were harvested.

### RNA interference

siRNAs targeting human RGC32 were screened to determine the optimum sequence. A sequence (5-GAUUCACUUUAUAGGAACATT-3) targeting human RGC32 was tested and used in our experiments. The siRNAs were synthesized and cloned into a pGPU6/GFP/Neo siRNA vector (Gene Pharma, Shanghai, People’s Republic of China). SW620 cells were transfected with siRNA using Lipofectamine 2000. The knockdown efficiency was confirmed by real-time RT-PCR and western blotting.

### *In vitro* cell growth assay

Cells were seeded in 96-well plates at density of 1 × 10^3^ cells/well and incubated for 1, 2, 3, 4 or 5 days. Cell proliferation was evaluated using a Cell Counting Kit-8 (CCK-8, Dojindo, USA) according to the manufacturer’s instructions. Briefly, 10 μl of CCK-8 solution was added to the culture medium and incubated for 2 h. The absorbance at a wavelength of 450 nm was measured, with a reference wave length of 650 nm. All experiments were repeated three times.

### Transwell *in vitro* invasion assays

An invasion assay was conducted using Transwell cell culture chambers (24 wells, 8 μm pore size; Corning). Briefly, upper inserts were coated with 50 μl of 10 mg/ml Matrigel (BD Biosciences) and allowed to set for 1 h at 37 °C. CRC cells were added to the top chamber, and the bottom chamber contained medium with 10% FBS as a chemoattractant. After 48 h, invasive cells were fixed and stained with Giemsa. Cells remaining on the upper surface of the insert membrane were removed with cotton swabs. The invasive cells were counted at 200x magnification in 5 different fields for each insert.

### Scratch wound-healing assay

Cells were seeded in 24-well plates at a density of 1 × 10^5^ cells/well and cultured under standard conditions until they reached 80–90% confluence. The cells were then treated with mitomycin C (10 μg/ml) during the wound healing assay. Cell migration was assessed by measuring the movement of cells into the acellular area created by a sterile insert. The wound closure was observed after 96 h.

### Tumorigenesis in nude mice

Four to six-week-old athymic BALB/c nude mice were obtained from the Experimental Animal Centre of Southern Medical University (permission number: SCXK2011-0015). All animal experiments were conducted such that the animals received ethical and humane treatment, in accordance with a license from the Guangdong Provincial Bureau of Science, and all procedures were approved by the Institutional Animal Care and Use Committee of Southern Medical University. For the *in vivo* tumor growth assay, after being re-suspended in serum-free medium, 5 × 10^6^ cells were injected subcutaneously into the left or right flank of nude mice (n = 6 per group). Three weeks later, tumors were removed and measured. Tumor volume was calculated as follows: volume = (a × b^2^)/2, where a meant the longest diameter and b meant the shortest diameter. The tumors were excised and fixed with 10% neutral-buffered formalin, and then, 4-μm-thick sections were cut. The sections were stained with haematoxylin and eosin according to standard protocols, and then, further IHC staining was performed using antibodies against Ki67, E-cadherin and vimentin.

### Statistical analysis

SPSS version 13.0 software was used for all statistical analyses. Comparisons between groups to assess statistical significance were performed with two-tailed paired Student’s t-tests. Chi-squared tests were used to analyse the correlation between the clinicopathological features of CRC and RGC32 expression. Survival curves were plotted according to the Kaplan–Meier method and compared log-rank tests. The significance of various survival-related variables was assessed with a Cox regression model in a multivariate analysis. *In vitro* cell growth was compared using one-way ANOVA. P < 0.05 was considered significant.

## Additional Information

**How to cite this article:** Wang, X.-Y. *et al*. RGC32 induces epithelial-mesenchymal transition by activating the Smad/Sip1 signaling pathway in CRC. *Sci. Rep.* **7**, 46078; doi: 10.1038/srep46078 (2017).

**Publisher's note:** Springer Nature remains neutral with regard to jurisdictional claims in published maps and institutional affiliations.

## Supplementary Material

Supplementary Results

## Figures and Tables

**Table 1:** Correlation between clinicopathological features and RGC32 expression.

Characteristics	Low, n (%)	High, n (%)	χ2 value	*P* value
Frequency (%)	13 (7.7)	170 (92.3)		
Sex, n (%)				
Male	46 (42.2)	63 (57.8)	0.251	0.616
Female	34 (45.9)	40 (54.1)		
Age, n (%)				
<50	59 (43.1)	78 (56.9)	0.094	0.76
≥50				
Tumor size (diameter in cm)				
<5	39 (39.8)	59 (60.2)	0.225	0.636
≥5	41 (43.2)	54 (56.8)		
Tumor differentiation				
Good	24 (40.0)	36 (60.0)	0.525	0.769
Moderate	40 (46.0)	47 (54.0)		
Poor	16 (44.4)	20 (55.6)		
Depth of tumor invasion				
Subserosa	7 (41.2)	10 (58.8)	4.932	0.085
Serosa	34 (37.0)	58 (63.0)		
Adjacent organs	40 (54.1)	34 (47.3)		
Lymph node status				
Negative	39 (52.7)	35 (47.3)	4.078	0.043
Positive	41 (24.4)	68 (75.6)		
Dukes classification				
A+B	42 (38.9)	66 (61.1)	4.446	0.035
C+D	41 (54.7)	34 (45.3)		
TNM stage				
stage I+II	41 (37.3)	69 (62.7)	3.908	0.048
stage III+IV	38 (52.1)	35 (47.9)		

## References

[b1] Torre L. A. . Global cancer statistics, 2012. CA: A Cancer Journal for Clinicians 65, 87 (2015). 25651787 10.3322/caac.21262

[b2] Hur K. . MicroRNA-200c modulates epithelial-to-mesenchymal transition (EMT) in human colorectal cancer metastasis. Gut 62, 1315 (2013). 22735571 10.1136/gutjnl-2011-301846 PMC3787864

[b3] Badea T. C., Niculescu F. I., Soane L., Shin M. L. & Rus H. Molecular cloning and characterization of RGC-32, a novel gene induced by complement activation in oligodendrocytes. J Biol Chem 273, 26977 (1998). 9756947 10.1074/jbc.273.41.26977

[b4] Kim D. S. . Promoter methylation of the RGC32 gene in nonsmall cell lung cancer. Cancer-Am Cancer Soc 117, 590 (2011). 10.1002/cncr.25451 20862745

[b5] Notaridou M. . Common alleles in candidate susceptibility genes associated with risk and development of epithelial ovarian cancer. Int J Cancer 128, 2063 (2011). 20635389 10.1002/ijc.25554 PMC3098608

[b6] Lu Y. & Hu X. B. C5a stimulates the proliferation of breast cancer cells via Akt-dependent RGC-32 gene activation. Oncol Rep 32, 2817 (2014). 25230890 10.3892/or.2014.3489

[b7] Xu R. . Knockdown of response gene to complement 32 (RGC32) induces apoptosis and inhibits cell growth, migration, and invasion in human lung cancer cells. Mol Cell Biochem 394, 109 (2014). 24833469 10.1007/s11010-014-2086-3

[b8] Eskandari-Nasab E., Hashemi M. & Rafighdoost F. Promoter Methylation and mRNA Expression of Response Gene to Complement 32 in Breast Carcinoma. J Cancer Epidemiol 2016, 7680523 (2016). 27118972 10.1155/2016/7680523 PMC4828546

[b9] Cui X. B., Luan J. N. & Chen S. Y. RGC-32 Deficiency Protects against Hepatic Steatosis by Reducing Lipogenesis. J Biol Chem 290, 20387 (2015). 26134570 10.1074/jbc.M114.630186 PMC4536444

[b10] Kruszewski A. M. . RGC-32 as a potential biomarker of relapse and response to treatment with glatiramer acetate in multiple sclerosis. Exp Mol Pathol 99, 498 (2015). 26407760 10.1016/j.yexmp.2015.09.007 PMC6594183

[b11] Li Z. . Response gene to complement 32 is essential for fibroblast activation in renal fibrosis. J Biol Chem 286, 41323 (2011). 21990365 10.1074/jbc.M111.259184 PMC3308844

[b12] Huang W. Y. . RGC-32 mediates transforming growth factor-beta-induced epithelial-mesenchymal transition in human renal proximal tubular cells. J Biol Chem 284, 9426 (2009). 19158077 10.1074/jbc.M900039200 PMC2666595

[b13] Browne G., Sayan A. E. & Tulchinsky E. ZEB proteins link cell motility with cell cycle control and cell survival in cancer. Cell Cycle 9, 886 (2010). 20160487 10.4161/cc.9.5.10839

[b14] Peinado H., Olmeda D. & Cano A. Snail, Zeb and bHLH factors in tumor progression: an alliance against the epithelial phenotype? Nat Rev Cancer 7, 415 (2007). 17508028 10.1038/nrc2131

[b15] Sheng G., Dos R. M. & Stern C. D. Churchill, a zinc finger transcriptional activator, regulates the transition between gastrulation and neurulation. Cell 115, 603 (2003). 14651851 10.1016/s0092-8674(03)00927-9

[b16] Rogers C. D., Saxena A. & Bronner M. E. Sip1 mediates an E-cadherin-to-N-cadherin switch during cranial neural crest EMT. J Cell Biol 203, 835 (2013). 24297751 10.1083/jcb.201305050 PMC3857483

[b17] Lander R., Nordin K. & LaBonne C. The F-box protein Ppa is a common regulator of core EMT factors Twist, Snail, Slug, and Sip1. J Cell Biol 194, 17 (2011). 21727196 10.1083/jcb.201012085 PMC3135407

[b18] Nitta K. R. . The N-terminus zinc finger domain of Xenopus SIP1 is important for neural induction, but not for suppression of Xbra expression. Int J Dev Biol 51, 321 (2007). 17554684 10.1387/ijdb.062252kn

[b19] van Grunsven L. A. . XSIP1, a Xenopus zinc finger/homeodomain encoding gene highly expressed during early neural development. Mech Dev 94, 189 (2000). 10842070 10.1016/s0925-4773(00)00318-x

[b20] Delalande J. M., Guyote M. E., Smith C. M. & Shepherd I. T. Zebrafish sip1a and sip1b are essential for normal axial and neural patterning. Dev Dyn 237, 1060 (2008). 18351671 10.1002/dvdy.21485 PMC2443937

[b21] Schlick S. N. . Upregulation of the cell-cycle regulator RGC-32 in Epstein-Barr virus-immortalized cells. Plos One 6, e28638 (2011). 22163048 10.1371/journal.pone.0028638 PMC3232240

[b22] Saigusa K. . RGC32, a novel p53-inducible gene, is located on centrosomes during mitosis and results in G2/M arrest. Oncogene 26, 1110 (2007). 17146433 10.1038/sj.onc.1210148

[b23] Vlaicu S. I. . Epigenetic modifications induced by RGC-32 in colon cancer. Exp Mol Pathol 88, 67 (2010). 19883641 10.1016/j.yexmp.2009.10.010 PMC2815209

[b24] Fosbrink M. . Overexpression of RGC-32 in colon cancer and other tumors. Exp Mol Pathol 78, 116 (2005). 15713436 10.1016/j.yexmp.2004.11.001

[b25] Conidi A. . Four amino acids within a tandem QxVx repeat in a predicted extended alpha-helix of the Smad-binding domain of Sip1 are necessary for binding to activated Smad proteins. Plos One 8, e76733 (2013). 24146916 10.1371/journal.pone.0076733 PMC3795639

[b26] Yoshida R. . Clinical Significance of SIP1 and E-cadherin in Patients with Esophageal Squamous Cell Carcinoma. Ann Surg Oncol 22, 2608 (2015). 25564163 10.1245/s10434-014-4314-1

[b27] Takeyama Y. . Knockdown of ZEB1, a master epithelial-to-mesenchymal transition (EMT) gene, suppresses anchorage-independent cell growth of lung cancer cells. Cancer Lett 296, 216 (2010). 20452118 10.1016/j.canlet.2010.04.008 PMC3110825

[b28] Kallergi G. . Epithelial to mesenchymal transition markers expressed in circulating tumor cells of early and metastatic breast cancer patients. Breast Cancer Res 13, R59 (2011). 21663619 10.1186/bcr2896 PMC3218948

[b29] Donninger H. . Whole genome expression profiling of advance stage papillary serous ovarian cancer reveals activated pathways. Oncogene 23, 8065 (2004). 15361855 10.1038/sj.onc.1207959

[b30] Liu Y. . An epigenetic role for PRL-3 as a regulator of H3K9 methylation in colorectal cancer. Gut 62, 571 (2013). 22345654 10.1136/gutjnl-2011-301059

