# Supplementary material for: RGC32 induces epithelial-mesenchymal transition by activating the Smad/Sip1 signaling pathway in CRC
Source: Sci Rep. 2017 May 4;7:46078. doi: 10.1038/srep46078 (PMC5415763; doi:10.1038/srep46078)
Supplement: Supplementary Results [file srep46078-s1.pdf]

RGC32 induces epithelial-mesenchymal transition by activation of the Smad/Sip1 signalling pathway in CRC

Xiao-Yan Wang[1,2]*, Sheng-Nan Li[1,2]*, Hui-Fang Zhu[1,2]*, Zhi-Yan Hu[1,2], Yan Zhong[1,2], Chuan-Sha Gu[1,2], Shi-You Chen[3], Teng-fei Liu [1,2], Zu-Guo Li [1,2]

1 Department of Pathology, Nanfang Hospital, Southern Medical University, Guangzhou 510515, China

2 Guangdong Provincial Key Laboratory of Molecular Tumour Pathology, Department of Pathology, School of Basic Medical Sciences, Southern Medical University, Guangzhou 510515, China

3 Department of Physiology & Pharmacology, University of Georgia, Athens, GA, United States

*****These authors contributed equally to this work

Corresponding authors:

1 Teng-Fei Liu, Department of Pathology, Nanfang Hospital, Southern Medical University, Guangzhou, China 510515, Email: liutengfei_1968@163.com

2 Zu-Guo Li, Department of Pathology, Nanfang Hospital, Southern Medical University, Guangzhou, China 510515, Email: lizg@smu.edu.cn

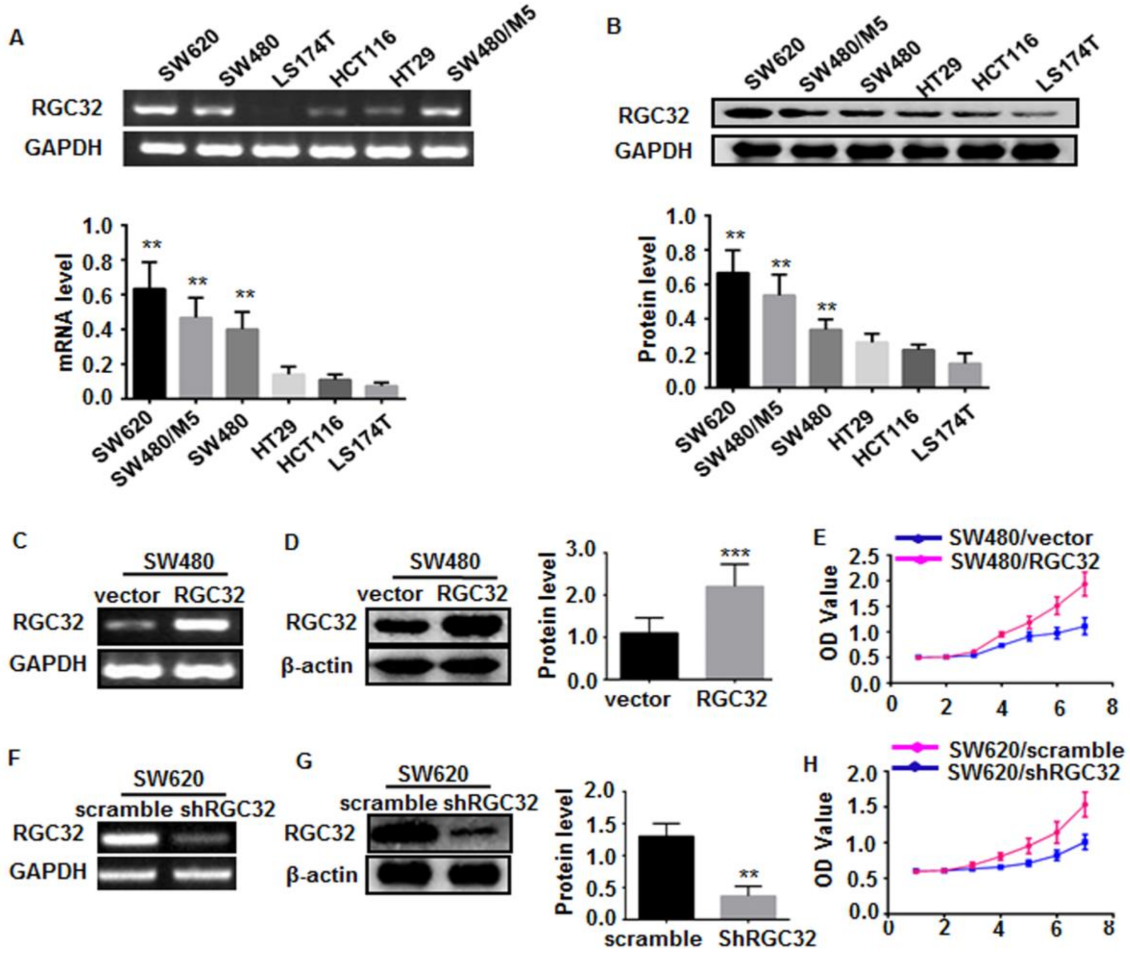

Supplement Figure 1. RGC32 promoted cell proliferation of CRC in vitro. (A) Expression of RGC32 mRNA in CRC cell lines. (B) Expression of RGC32 protein in CRC cell lines. **, $P < 0.01$ vs LS174T. (C, D) RT-PCR and western blot analysis of RGC32 expression in SW480 cells treated with vector or RGC32 plasmid (RGC32). ***, $P < 0.001$ vs vector. (E) The proliferation of SW480 cells was increased after RGC32 expression was up-regulated. (F, G) RT-PCR and western blot analysis of RGC32 expression in SW620 cells treated with scramble or RGC32 shRNA (shRGC32). (H) The proliferation of SW620 cells was decreased after RGC32 expression was down-regulated. **, $P < 0.01$ vs scramble.

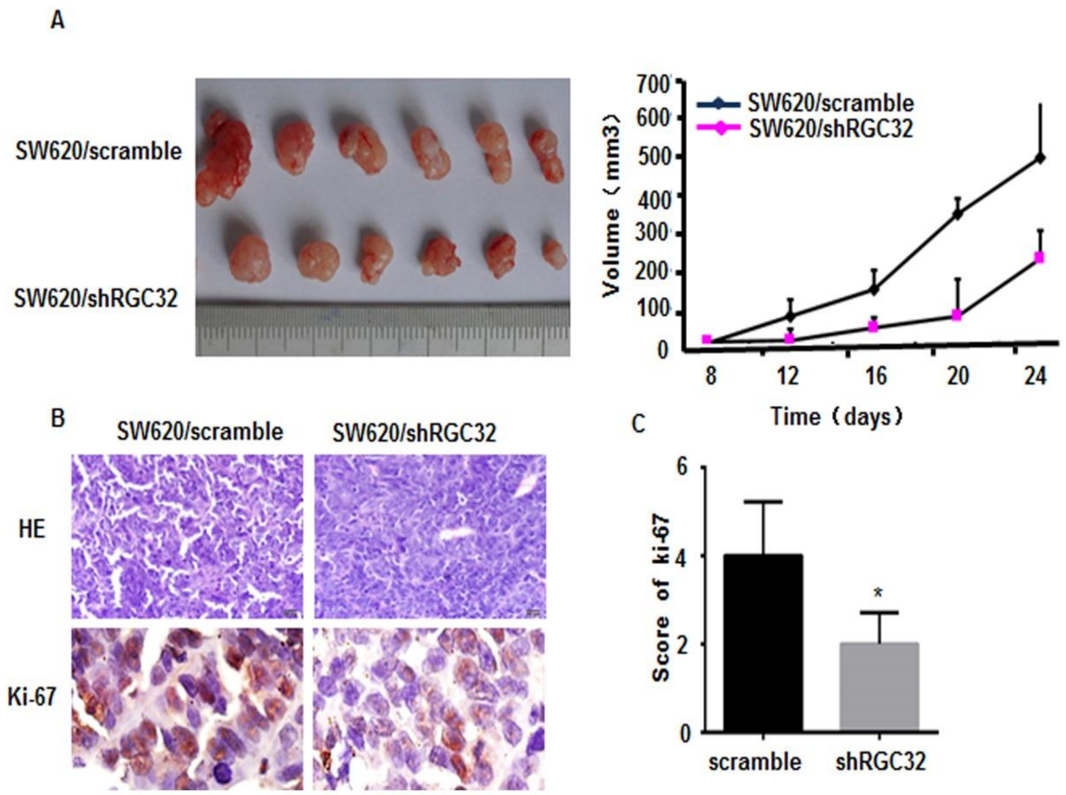

Supplement Figure 2. Knockdown of RGC32 inhibited tumor growth in vivo. SW620 cells transduced with scramble or RGC32 shRNA (shRGC32) were injected subcutaneously into nude mice. (A) Tumors were removed 24 days after injection and imaged. n = 6. Tumor derived from SW620-attenuated RGC32 grew significantly lower than that from the scramble-treated cells. (B) Representative photographs of H&E and Ki-67 immunohistochemical staining of xenograft tumors. The Ki-67 index in shRGC32-treated cells was significantly lower than that of scramble-treated cells. (C) Quantification of Ki-67 expression. *P<0.05 compared to the scramble-treated group, n = 6.
